# Cytosolic Nuclear Sensor Dhx9 Controls Medullary Thymic Epithelial Cell Differentiation by p53-Mediated Pathways

**DOI:** 10.3389/fimmu.2022.896472

**Published:** 2022-06-03

**Authors:** Xue Dong, Jiayu Zhang, Qian Zhang, Zhanfeng Liang, Yanan Xu, Yong Zhao, Baojun Zhang

**Affiliations:** ^1^ State Key Laboratory of Membrane Biology, Institute of Zoology, Chinese Academy of Sciences, Beijing, China; ^2^ University of Chinese Academy of Sciences, Beijing, China; ^3^ Beijing Institute for Stem Cell and Regeneration, Beijing, China; ^4^ Department of Pathogenic Microbiology and Immunology, School of Basic Medical Sciences, Xi’an Jiaotong University, Xi’an, China

**Keywords:** thymus, thymic epithelial cells, T cell development, P53, cell cycle arrest, immune tolerance

## Abstract

Thymic epithelial cells (TECs) critically participate in T cell maturation and selection for the establishment of immunity to foreign antigens and immune tolerance to self-antigens of T cells. It is well known that many intracellular and extracellular molecules elegantly have mastered the development of medullary TECs (mTECs) and cortical TECs (cTECs). However, the role played by NTP-dependent helicase proteins in TEC development is currently unclear. Herein, we created mice with a TEC-specific DExD/H-box helicase 9 (*Dhx9*) deletion (*Dhx9* cKO) to study the involvement of Dhx9 in TEC differentiation and function. We found that a Dhx9 deficiency in TECs caused a significant decreased cell number of TECs, including mTECs and thymic tuft cells, accompanied by accelerated mTEC maturation but no detectable effect on cTECs. Dhx9-deleted mTECs transcriptionally expressed poor tissue-restricted antigen profiles compared with WT mTECs. Importantly, *Dhx9* cKO mice displayed an impaired thymopoiesis, poor thymic T cell output, and they suffered from spontaneous autoimmune disorders. RNA-seq analysis showed that the Dhx9 deficiency caused an upregulated DNA damage response pathway and Gadd45, Cdkn1a, Cdc25, Wee1, and Myt1 expression to induce cell cycle arrest in mTECs. In contrast, the p53-dependent upregulated RANK-NF-κB pathway axis accelerated the maturation of mTECs. Our results collectively indicated that Dhx9, a cytosolic nuclear sensor recognizing viral DNA or RNA, played an important role in mTEC development and function in mice.

## Introduction

Thymic epithelial cells (TECs) are the most important stromal cells in thymic microenvironments in regard to imposing self/non-self-recognition of T cells. Cortical thymic epithelial cells (cTECs) and medullary thymic epithelial cells (mTECs) construct a three-dimensional epithelial network corporately, which supports the stepwise development of thymocytes and the generation of thymic regulatory CD4^+^CD25^+^ T cells (Treg) (1–3). The early thymic progenitors (ETPs) migrate from the cortico-medullary junction (CMJ) into the thymus, where they undergo lineage commitment and positive selection driven by cTECs (4, 5). Positive selected thymocytes then go through negative selection in the medulla region, and thymocytes with high affinity to self-antigens are deleted (5, 6). It is known that mTECs are a heterogeneous population (7). According to the expression level of CD80 and MHCII, mTECs can be sub-divided into CD80^+^MHCII^hi^ (mTEC^hi^) and CD80^-^MHCII^lo^ (mTEC^lo^). Negative selection in the medulla largely relies on Aire^+^ mTEC^hi^ cells, which express and present thousands of tissue-restricted antigens (TRAs) (8). Recently, terminal differentiated DCLK1^bright^ mTECs has been identified as thymic tuft cells (9, 10). There is a great similarity between thymic and small-intestinal tuft cells, and thymic tuft cells are important to the development of thymus-resident type-2 innate lymphoid cells (ILCs) and thymic invariant natural killer T cells (iNKTs) (11).

The development and maturation of mTECs involve a complicated regulatory network of external and internal signals. Many studies have provided great insights into the regulation of the TNFR superfamily (TNFRSF) in mTECs, such as the receptor activator for NFκB (RANK), CD40, and lymphotoxin β receptor (LtβR) (12, 13). Mice bearing RANK, CD40, or LtβR deficiency exhibited different levels of retardation in mTEC development and maturation (12, 14). Moreover, both the canonical and non-canonical NF-κB pathways integrating signals from multiple TNFRSF subsequently initiate the transcriptional programs, the disruption of which lead to impaired medulla organization and severe autoimmunity, including NIK, IKKa, RelB, and TRAF6 (15–17). In addition, it was reported that the tumor suppressor protein p53 functioned as a molecular hub in mTECs, so p53 cKO mice had a damaged RANK-driven maturation of mTECs in the thymus (18). Notably, the inactivation of Dicer in TECs unveiled the pivotal role of microRNAs in governing TEC development and maturation (19). However, the regulatory role played by helicase proteins in TECs is still unknown.

DExD/H-box helicase 9 (Dhx9) is recognized as a cytosolic nuclear sensor recognizing viral DNA or RNA, and it plays an important role in host antiviral innate immunity (20, 21). Dhx9, also known as RNA helicase A (RHA), is an NTP-dependent helicase protein capable of unwinding DNA and RNA duplexes, as well as more complex nucleic acid structures (22–24). Structurally, Dhx9 is a multi-domain protein, including a conserved helicase core domain, two double-stranded RNA-binding domains, a nuclear transport domain, and a single-stranded DNA-binding RGG-box (25). Dhx9, with a large number of interacting partners, appears to participate in many cellular processes, such as DNA replication, transcription, translation, microRNA biogenesis, RNA processing and transport, and the maintenance of genomic stability (22). It was reported that *Dhx9* knockdown by shRNA in human diploid fibroblasts could block DNA replication in a p53-dependent manner, which led to growth arrest and senescence (26). Otherwise, Dhx9 also regulated the antiviral infection capacity of CD8^+^ T cells or macrophages in a way that was independent of nuclear sensor function (24, 27). Furthermore, Dhx9 was considered as a candidate target for cancer therapy (28). Herein, we created mice with a TEC-specific Dhx9 deletion and studied the role of Dhx9 in TEC differentiation. Our results showed that Dhx9 deletion in TECs resulted in severe thymus atrophy with the accelerated mTEC maturation, central immune tolerance disruption, and spontaneous development of autoimmune diseases. Thus, we conclude that Dhx9 plays an indispensable role in the development and maturation of mTECs and in the establishment of central immune tolerance.

## Materials and Methods

### Mice

Our scientific research was performed using the TEC-conditional homozygous Dhx9-deleted mice model. We recombined the Cre-loxp system in TECs *via* crossing the *Dhx9* ^loxp/loxp^ mice to the Foxn1^Cre^ mice. We used the *Dhx9* ^loxp/loxp^ littermates as WT controls. All mice were kept under specific pathogen-free conditions. Age-matched mice displayed a single representative experiment in figures. The *Dhx9* ^loxp/loxp^ mice came from Dr. Baojun Zhang, Department of Pathogenic Microbiology and Immunology, School of Basic Medical Sciences, Xi’an Jiaotong University (Xi’an, Shaanxi, China) (24). The Foxn1^Cre^ mice were provided by Dr. Yu Zhang of Peking University, Health Science Center (Beijing, China). With the improvement of the Animal Ethics Committee of the Institute of Zoology (Beijing, China), all animal experiments were operated under the guidance of the ethical care and use of laboratory animals.

### Purification of TECs

TECs are an extreme low frequency cell population in thymus. We enriched TECs through free sedimentation, combined with magnetic-activated cell sorting (MACS) or fluorescence-activated cell sorting (FACS). Then 4-week-old mice were euthanized to collect the thymic lobes. After being washed twice in cold PBS, the thymic lobes were immediately cut into homogeneous tiny pieces and collected with Dulbecco’s Modified Eagle’s Medium (DMEM), containing 2% FBS into 15 mL numbered centrifuge tubes. The dissociative thymocytes were released into DMEM while the tissue fragments settled freely to the bottom of the tube as thymic stroma. To acquire single cell suspension, the tissue fragments were resuspended with DMEM, containing 2% FBS, 1 mg/mL collagenase/dispase (11097113001; Sigma-Aldrich), and 20 U/mL DNAse I (D5025; Sigma-Aldrich) and then incubated at 37°C for 45 min. When the tissue pieces disappeared, we used 5 mL of PBS containing 1% FBS and 5 mM EDTA to neutralize cell mixture gently, which was blown several times and filtered with a 200-mesh filter to obtain single-cell suspension. The cells were then centrifuged, resuspended in DMEM (containing 2% FBS), and counted for further experiments. As for MACS, anti-mouse CD45 microbeads (Miltenyi Biotec, 130052301) were used to further TEC enrichment, according to the supplier’s protocols.

### Flow Cytometry and Antibodies

For flow cytometry analysis, 1×10^6^ fresh isolated cells were resuspended with 100 μL FACS buffer (PBS containing 0.1% BSA, 0.02% NaN_3_) and incubated with fluorescein conjugated antibody mixture in 4 °C for 30 min. Anti-CD16/32 antibody (12-0161-82, clone 93) and fixable viability dye eFluor™ 506 (65–0866–18) are indispensable for blocking the Fc receptor and eliminating dead cells. The fixation buffer (eBioscience, 00-5123-43 and 00-5223-56) and permeabilization buffer were used to fix and permeabilize cells for intracellular staining, such as Aire, Ki67, Helios, Foxp3, RORγt, and PLZF. For the detection of thymic tuft cells, the secondary antibody Alexa Fluor 647-conjugated donkey anti-rabbit IgG (H+L) (Jackson ImmunoResearch Laboratories, 711-605-152) was used after intracellular staining of DCAMKL1 (DCLK1) (Abcam, ab31704). All operations were shielded from light at 4 °C until analyzed by flow cytometry.

According to the identification of different cell populations, the fluorochrome-conjugated antibodies used in the experiment were listed below. For TECs, we used CD45 (Biolegend; 103132; clone 30-F11), EpCAM (Biolegend; 118215; clone G8.8), Fluorescein-labeled Ulex Europaeus Agglutinin I (UEA1) (FL-1061; Vector Laboratories), Ly51 (Biolegend; 108312; clone 6C3), Ly51 (BD Biosciences; 740882; clone BP-1), CD40 (Biolegend; 124610; clone 3/23), I-A/I-E (Biolegend; 107632; clone M5/114.15.2), CD80 (eBioscience; 12-0801-82; clone 16-10A1), and Aire (eBioscience; 50-5934-80; clone 5H12). For conventional T cells, we used TCRβ (Biolegend; 109222; clone H57-597), CD4 (Biolegend; 100414; clone GK1.5), CD8 (Biolegend; 100738; clone 53-6.7), CD19 (Biolegend; 152404; 1D3/CD19), CD11b (eBioscience; 11-0112-85; clone M1/70), F4/80 (eBioscience; 11-4801-82; clone BM8), NK1.1 (eBioscience; 11-5941-81; clone PK136), CD44 (Biolegend; 103055; clone IM7), CD279 (Biolegend; 135216; clone 29F.1A12), CD5 (Biolegend; 100625; clone 53-7.3), Ter119 (Biolegend; 116206; clone TER-119), CCR7 (Biolegend; 120106; clone 4B12), Foxp3 (eBioscience; 11-5773-82; clone FJK-16s), CD25 (eBioscience; 12-0251-82; clone PC61.5), CD24 (eBioscience; 11-0242-82; clone M1/69), CD69 (eBioscience; 48-0691-82; clone H1.2F3), CD62L (eBioscience; 12-0621-82; clone MEL-14), and CD45RB (eBioscience; 11-0455-82; clone C363.16A). For the iNKT cells, we used PLZF (eBioscience; 53-9320-80; clone Mags.21F7), RORγt (eBioscience; 562894; clone Q31-378), and CD1d tetramer (ProImmune; D001-2X). For the kit, we used the PE Active Caspase-3 Apoptosis Kit (BD Biosciences; 550914). We performed a flow cytometric detection with a Gallios Flow Cytometer (Beckman Coulter, United States) or a BD LSRFortessa X-20 Flow Cytometer (BD Biosciences, United States) and analyzed the results with FlowJo software (BD Biosciences).

### TECs Cultured *In Vitro*

TECs have the ability to proliferate when cultured in Thymic Epithelial Cell Medium (TyEpiCM, ScienCell Research Laboratories, Catalog #3911). We collected the thymic lobes from neonatal WT and *Dhx9* cKO mice, respectively, and cut them into homogeneous tiny pieces, as described above. But, unlike in “Purification of thymic epithelial cells”, the small pieces were resuspended by TyEpiCM directly and incubated in a sterilized condition of 37°C with 5% CO_2_ for 7 days. In the meantime, we washed the culture dishes with 37°C PBS and changed the medium every other day to remove nonadherent thymocytes and the dead cells.

### Cell Cycle Analysis

Cell cycle distribution was determined by a BD LSRFortessa X-20 Flow Cytometer and analyzed by a “cell cycle” module in FlowJo software. Generally, cultured TECs were disassociated by trypsin EDTA 1× (25-053-CI, Corning) at 37°C for 5-10 min, collected into 5 mL polystyrene round-bottom tube and washed twice with cold PBS. Cells were fixed in 75% ethanol overnight at -20°C and followed by washing twice with 3-5 mL PBS. The fixed cells were then incubated with 100 μL 100 μg/mL RNaseA and 25 μL 100 μg/mL propidium iodide (PI) for 30 min at room temperature.

### Hematoxylin and Eosin (H&E) Straining

In fact, 4% paraformaldehyde fixed, paraffin embedded tissue sections (6 µm) were deparaffinized in xylene and rehydrated in an ethanol series. Hematoxylin and eosin (H&E) staining was directed by the reported standard protocol. Finally, these slides were captured by Leica Aperio VESA8. Inflammatory cell infiltration has five levels, as previously reported (29, 30).

### Immunofluorescence Staining

Thymus, embedded with the optimal cutting temperature compound, was cut into 6 μm slides. The slides or cells were fixed for 20 min with 4% polyoxymethylene and blocked in PBS containing 1% BSA. Immunofluorescence staining was performed by the reported standard protocol (31). For analysis of the thymic medulla and cortex region, the thymic slides were strained *via* primary antibodies [rabbit anti-KRT5 (Covance; PRB-160P; clone AF 138) and rat anti-KRT8 (DSHB; ab531826; Troma-I)] and followed by the secondary antibodies Alexa Fluor 594-conjugated donkey anti-rabbit IgG (H+L) (Jackson ImmunoResearch Laboratories; 711-586-152) and Alexa Fluor 488-conjugated donkey anti-rat IgG (H+L) (Jackson Immuno Research Laboratories; 712-546-150). For the detection of antinuclear antibodies in 8-month-old WT and *Dhx9* cKO mice, Sera were diluted by 1:30 as primary antibodies. The combination was detected *via* Alexa Fluor 488-conjugated donkey anti-mice IgG (H + L) antibodies (Jackson Immuno Research Laboratories; 715-546-150). Nuclei were stained with 4’6-diamidino-2-phenylindole (DAPI; Sigma-Aldrich; D9542). Images were taken by a laser-scanning N-SIM super-resolution confocal microscope (Nikon, Tokyo, Japan).

### Quantitative PCR With Reverse Transcription (RT-qPCR)

The MicroElute Total RNA Kit (Omega Bio-Tek, R6831) or TRIzol Reagent (Invitrogen, 15596-018) were used to extract total RNA from the sorted TECs, according to the manufacturer’s instructions. Next, unstable mRNA was reversely transcribed into cDNA by the SuperScript III Reverse Transcriptase Kit (Invitrogen, 18080-093). Using SYBR Premix Ex TaqTM (TaKaRa, RR420), a quantitative PCR of target genes was performed on a CFX96 apparatus (Bio-Rad Laboratories). For data analysis, expressions of target genes were normalized by *Hprt* using the 2-ΔΔCt method. All primers used in this study were *Hprt*-forward 5′-TGAAGAGCTACTGT-AATGATCAGTCAA-3′, *Hprt*-reverse 5′-AGCAAGCTTGCAACCTTAACCA-3′, *Dhx9*-forward 5′-CCACCCATACTTAGCGACAC-3′, and *Dhx9*-reverse 5′-CCATAGCCAGAAGACTCAACC-3′.

### Bulk RNA-Seq Sample Preparation and Analysis

Single-cell suspensions of mTEC^hi^ and mTEC^lo^ from WT and *Dhx9* cKO mice were sorted using a Fusion cell sorter (BD Biosciences), with two independent parallel samples in each group (**Figure S2**). The operations are consistent with the previously published method (32). We used DEGseq to identify the differential expressed genes (q-value < 0.05, |log_2_FC| > 0) of mTEC^hi^ and mTEC^lo^ from the different genotype mice, respectively (33). According to the differential expressed genes in mTECs of WT and *Dhx9* cKO mice, GSVA was performed to calculate the individual gene set enrichment scores, which were visualized by R3.6.0. The analysis of the KEGG pathway was performed by KOBAS 3.0 (34). GSEA was carried out by searching the KEGG database (34). All analyses were carried out with *P* < 0.05 as the cutoff criterion. The raw data of RNA-seq sequencing used in this article could be found in the National Genomics Data Center (NGDC), part of the China National Center for Bioinformation (CNCB) under number PRJCA008466.

### Statistical Analysis

We used a student’s unpaired *t*-test for the statistical analysis of the WT and *Dhx9* cKO mice. Sample sizes for the statistical analysis were at least *n* = 3, and the statistical significant *P*-value was taken as *P* < 0.05. Errors were shown as standard deviations (SD) throughout. There were no limitations on the repeatability of the experiments, and no samples were excluded from this analysis.

## Results

### Inactivation of Dhx9 in TECs Remarkably Hindered mTEC Development

**Figure 1:**
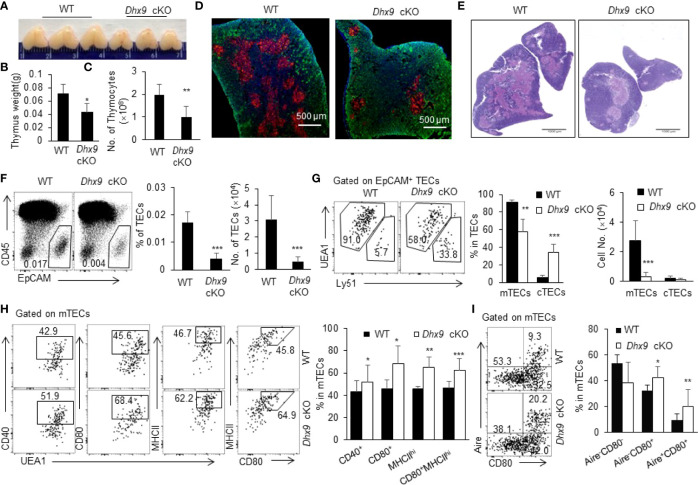
Deletion of Dhx9 in TECs compromised the development and maturation of TECs. **(A–C)** The representative pictures **(A)**, weight **(B)** and cell numbers **(C)** of the thymus isolated from 4-week-old WT and *Dhx9* cKO mice (*n* = 10 for each group). **(D)** Immunofluorescence staining of frozen thymic sections of WT and *Dhx9* cKO mice. Red: KRT5-marked medullary regions; Green: KRT8-marked cortical regions; Scale bars: 500 μm. **(E)** The representative thymic H&E staining in 4-week-old WT and *Dhx9* cKO mice; Scale bars: 1000 μm. **(F)** Flow cytometric profiles, frequencies, and cell numbers of CD45^-^EpCAM^+^ TECs in 4-week-old WT and *Dhx9* cKO mice (*n* = 7 for each group). **(G)** Representative flow cytometry plots, frequencies, and cell numbers of UEA1^+^Ly51^-^ mTECs and UEA1^-^Ly51^+^ cTECs in 4-week-old WT (*n* = 7) and *Dhx9* cKO mice (*n* = 6). **(H)** Representative flow cytometry plots and frequencies showed the maturation of mTECs, as measured by the expression of CD40, CD80, and MHCII in 4-week-old WT (*n* = 8) and *Dhx9* cKO mice (*n* = 6). **(I)** Representative flow cytometry plots and frequencies exhibited the maturation stage by the expression of CD80 and Aire in 4-week-old WT (*n* = 8) and *Dhx9* cKO mice (*n* = 6). One representative histogram represented the mean ± SD. The unpaired, two-tailed student’s *t*-test was used. *P < 0.05, **P < 0.01, ***P < 0.001 compared with WT control mice.

The *Dhx9* knock-out mouse model exhibited an embryonic lethal feature (35). Focusing on the specific regulation of Dhx9 in TECs, we generated mice with conditional Dhx9 deletion in TECs (*Dhx9* ^flox/flox^ and Foxn1^Cre^ *Dhx9* ^flox/flox^, designated as WT and *Dhx9* cKO mice, respectively) (**Figure S1A**). We detected the Cre-mediated specific deletion of *Dhx9* in TECs *via* real time PCR and confirmed the effective knockout of *Dhx9* in TECs rather than in thymocytes from *Dhx9* cKO mice (**Figure S1B**). *Dhx9* cKO mice were born without obvious abnormalities compared to WT littermates. We first evaluated the effect of *Dhx9* deficiency in the thymus morphologically and found that *Dhx9* cKO mice possessed a more shrunken thymus, embodied in a significant decreased thymus weight and the total cell number of thymocytes (**Figures 1A–C**). We next examined the cytokeratin 8 (KRT8) and cytokeratin 5 (KRT5)- immunofluorescence-stained sections of the thymus from 4-week-old WT littermates and *Dhx9* cKO mice. We observed that the medullary regions, highlighted by KRT8 (red), showed a remarkable decreased in *Dhx9* cKO mice, whereas the cortical region had an increased tendency in turn (**Figure 1D**). The similar phenotype was shown by the hematoxylin and eosin strain (H&E strain), with a shrunken medulla region in *Dhx9* cKO mice, compared with WT littermates (**Figure 1E**). These results showed that Dhx9 might play an important role in the formation of thymic medullary regions. Subsequently, we appraised the endogenous effect of Dhx9 on TEC subsets *via* flow cytometry analysis. TEC-specific inactivation of Dhx9 heavily reduced the percentage and cell number of CD45^-^EpCAM^+^ TECs in *Dhx9* cKO mice compared with WT littermates (**Figure 1F**). TECs are subdivided into cTECs and mTECs based on the disparate expression spectrum of specific markers, including Ly51 and UEA1 (2). Flow cytometry analysis unveiled that *Dhx9* cKO mice represented a measurable reduced percentage of mTECs but increased the percentage of cTECs (**Figure 1G**). More conspicuously, the cell number of mTECs in *Dhx9* cKO mice was cut down to one-tenth of those in WT littermates, whereas the cell number of cTECs in *Dhx9* cKO mice was similar to that of WT littermates (**Figure 1G**). Furthermore, we examined the percentage and cell number of TECs, mTECs and cTECs in 1-week-old WT and *Dhx9* cKO mice (**Figures S4A–D**). The results exhibited a significant reduced percentage and cell number of TECs (**Figures S4A, B**) and mTECs (**Figures S4C, D**), but not of cTECs, which was similar with the altered phenotypes detected in 4-week-old *Dhx9* cKO mice. These results collectively indicated that Dhx9 mignt regulate TEC development intrinsically and the obvious thymic atrophy in *Dhx9* cKO mice was mainly attributed by the hindered development of mTECs.

### Dhx9 Ablation in TECs Facilitates mTEC Maturation

We were also interested in the effects of Dhx9 on the differentiation and maturation of mTECs. The competent mature mTECs are capable of expressing a variety of TRAs randomly and facilitating the establishment of the central immune tolerance by eliminating self-reactive thymocytes (5, 36). In contrast with immature mTECs, the maturation process of mTECs is accompanied by elevated expressions of several key genes, such as CD40, CD80, and MHCII (37, 38). Pursuantly, we scrutinized the expression of CD40, CD80, and MHCII on mTECs. As shown in **Figure 1H**, TEC-specific *Dhx9* deletion significantly accelerated the maturation process of mTECs, as illustrated by the remarkable increasing proportion of CD40^+^, CD80^+^, and MHCII^hi^ mTEC populations (**Figures 1H** and **S4E**). According to MHCII and CD80 expressed on mTECs, two diacritical cell subsets were generally defined as MHCII^lo^CD80^-^ (mTEC^lo^) and MHCII^hi^CD80^+^ (mTEC^hi^) (**Figure S2**) (3). Flow cytometry analysis exhibited an apparent increased proportion of mTEC^hi^ in *Dhx9* cKO mice compared to WT littermates (**Figures 1H** and **S4F**). Previous studies have proved that Aire, as an indispensable transcriptional modulator for promiscuous gene expression of TRAs, is primarily expressed in the highly maturated mTEC population (39–41). Consistently, the percentage of intermediate mature Aire^-^CD80^+^ and mature Aire^+^CD80^+^ mTECs from *Dhx9* cKO mice increased significantly compared with WT littermates, whereas the change of unmatured Aire^-^CD80^-^ mTECs exhibited a contrary tendency when Dhx9 was deleted in TECs (**Figures 1I** and **S4G**). However, the cell number of mature mTECs marked by CD40, CD80, MHCII, and Aire reduced significantly in *Dhx9* cKO mice (**Figure S3**) because of the decreased total TEC cell number in these mice. Thus, Dhx9 deficiency in TECs accelerated the maturation process of mTECs noticeably.

### Thymopoiesis Is Compromised in *Dhx9* cKO Mice

**Figure 2:**
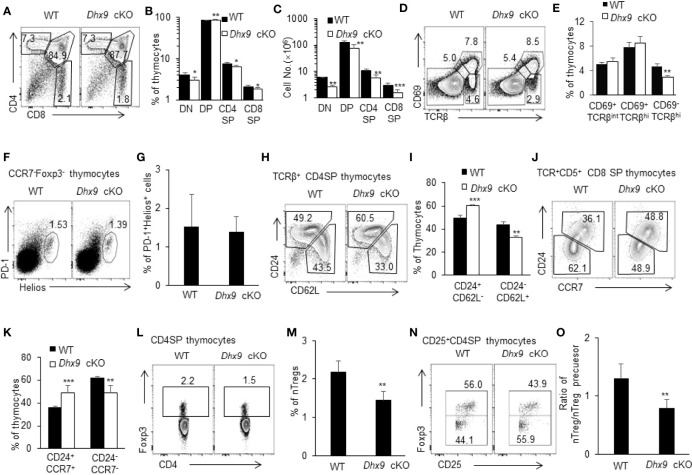
Dhx9 deletion in TECs hindered the development of thymocytes. **(A)** Representative flow cytometry plots for DN (CD4^-^CD8^-^), DP (CD4^+^CD8^+^), CD4SP (CD4^+^CD8^-^), and CD8SP (CD4^-^CD8^+^) populations in the thymus from 4-week-old WT and *Dhx9* cKO mice. **(B, C)** Frequencies **(B)** and cell numbers **(C)** of DN, DP, CD4SP, and CD8SP thymocytes of 4-week-old WT and *Dhx9* cKO mice. **(D, E)** Representative flow cytometry plots and frequencies **(E)** of CD69 and TCR β-chain expressed on total thymocytes from 4-week-old WT and *Dhx9* cKO mice. **(F, G)** Representative flow cytometry plots and frequencies **(G)** of Helios^+^PD-1^+^ thymocytes gated on TCRβ^+^CD25^-^CD4SP of 4-week-old WT and *Dhx9* cKO mice. **(H, I)** Representative flow cytometry plots and frequencies **(I)** of post-selection maturation measured by CD24 and CD62L expression in TCRβ^hi^CD4^+^CD8^-^ thymocytes from 4-week-old WT and *Dhx9* cKO mice. **(J, K)** Representative flow cytometry plots and frequencies **(K)** of post-selection maturation, as measured by CD24 and CCR7 expression in TCRβ^hi^CD5^+^CD4^-^CD8^+^ thymocytes from 4-week-old WT and *Dhx9* cKO mice. **(L, M)** Foxp3 expression by CD4^+^CD8^−^TCR^+^ thymocytes in 4-week-old WT and *Dhx9* cKO mice. **(N)** Representative flow cytometry plots showed the expression of Foxp3 on CD4^+^CD8^-^CD25^+^ thymocytes. **(O)** The histogram represented the ratio of nTreg (CD4^+^CD8^-^CD25^+^Foxp3^+^) to nTreg precursors (CD4^+^CD8^-^CD25^+^Foxp3^-^) for WT and *Dhx9* cKO mice. One representative histogram represented the mean ± SD and WT = 5, *Dhx9* cKO = 5 for each experiment. The unpaired, two-tailed student’s *t*-test was used. *P < 0.05, **P < 0.01, ***P < 0.001 compared with WT control mice.

MTECs and cTECs collectively provide a specialized thymus microenvironment for positive selection and negative selection to support the development of an immunocompetent and self-protective T cell pool (31, 32, 42). We next analyzed the thymopoiesis in the thymus from 4-week-old WT and *Dhx9* cKO mice. Distinguished by the expression of CD4 and CD8, thymocytes in *Dhx9* cKO mice showed a significant decreased percentage of CD4^+^CD8^-^ (CD4SP) and CD4^-^CD8^+^ (CD8SP) T cells, coupled with an increased percentage of CD4^+^CD8^+^ T cells (DP) compared to WT littermates (**Figures 2A, B**). Together with an overall decreased cell number of DN, DP, CD4SP, and CD8SP T cells in *Dhx9* cKO mice (**Figure 2C**), these results demonstrated that TEC-specific Dhx9 inactivation hindered the intra-thymic T cell development. The expression patterns of CD69 and TCRβ describe a differentiation process from DP into SP thymocytes (31, 43, 44). This process was impaired in *Dhx9* cKO mice, with a decreased percentage of CD69^+^TCRβ^hi^ thymocytes, in line with the reduced percentage and cell number of SP thymocytes (**Figures 2D, E**). As for the clonal deletion of Helios^+^PD-1^+^CCR7^-^Foxp3^-^ self-reactive thymocytes during negative selection (45), there was no significant difference between WT and *Dhx9* cKO mice (**Figures 2F, G**).

Before the export of the negative-selected SP thymocytes to peripheral T cell pool as recent thymic emigrants (RTEs), the non-fully matured SP thymocytes further undergo post-selection maturation, including the downregulation of both CD24 and CCR7, and the upregulation of CD62L (30, 46, 47). Further analysis demonstrated a blocked CD4SP maturation process, with a significant decreased percentage of mature TCRβ^+^CD62L^+^CD24^-^CD4SP thymocytes in *Dhx9* cKO mice and an increased proportion of the immature subset, TCRβ^+^CD62L^-^CD24^+^CD4SP (**Figures 2H, I**). Similarly, the downregulation of CD24 and CCR7 during the CD8SP maturation process was also blocked in *Dhx9* cKO mice, in contrast with WT littermates (**Figures 2J, K**). Moreover, TECs also critically support the development of nTregs from self-reactive CD4^+^ SP thymocytes whose TCR signal strength is not enough to be clonal deleted and drives the development of CD25^+^Foxp3^-^ nTreg precursors into mature CD25^+^Foxp3^+^ nTregs (48). Dhx9-deleted TECs lead to a decreased frequency of CD4^+^Foxp3^+^ nTregs (**Figures 2L, M**) and the lower ratio of CD25^+^Foxp3^+^ nTreg-to-CD25^+^Foxp3^-^ nTreg precursors (**Figures 2N, O**), indicating that the maturation of nTregs was hindered in the thymus of *Dhx9* cKO mice.

### Dhx9 Is Indispensable for the Differentiation of Thymic Tuft Cells

**Figure 3:**
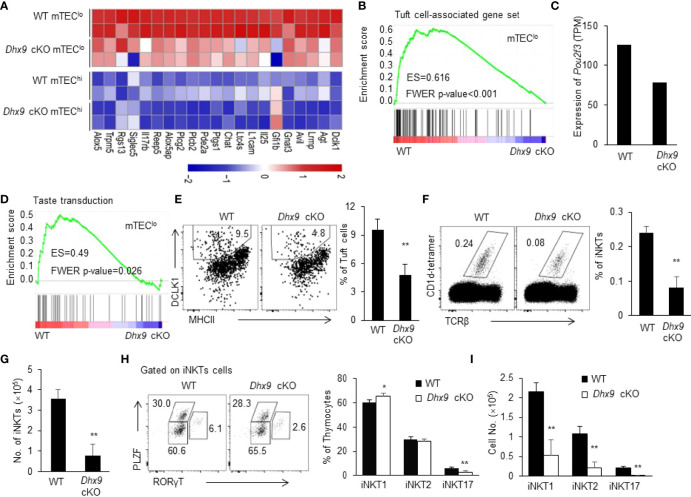
Dhx9 deficiency in TECs severely affected the development of thymic tuft cells. **(A)** The heatmap showed the differentially expressed genes in the bulk RNA-seq data from 4-week-old WT and *Dhx9* cKO mice that were highly expressed in thymic tuft cells. **(B)** The GSEA analysis of the Tuft cell-associated gene set between WT and Dhx9-deficient mTEC^lo^; *P* < 0.05. **(C)** The expression of *Pou2f3* in mTEC^lo^ from 4-week-old WT and *Dhx9* cKO mice from the bulk RNA-seq data. **(D)** GSEA showed the taste transduction pathway gene set enriched in *Dhx9* cKO mTEC^lo^; *P* < 0.05. **(E)** The representative flow cytometry plots (left) and percentage (right) of the thymic tuft cells were distinguished by the expression of MHCII and DCLK1 between 4-week-old WT and *Dhx9* cKO mice (*n* = 5 for each group). **(F)** Representative flow cytometry plots and percentages of iNKT cells in 4-week-old WT and *Dhx9* cKO mice (*n* = 5 for each group). **(G)** Cell number of iNKT cells in 4-week-old WT and *Dhx9* cKO mice (*n* = 5 for each group). **(H)** The distinct cell population of iNKT1, iNKT2, and iNKT17 between 4-week-old WT and *Dhx9* cKO mice (*n* = 5 for each group) were analyzed *via* flow cytometry. **(I)** Number of iNKT1, iNKT2, and iNKT17 in 4-week-old WT and *Dhx9* cKO mice (*n* = 5 for each group). One representative histogram represented the mean ± SD. The unpaired, two-tailed student’s *t*-test was used. *P < 0.05, **P < 0.01 compared with WT control mice.

With the application of single-cell genomic omics and fate mapping technologies, the conception of mTEC heterogeneity expands in terms of molecular function and development (7). IL25^+^ thymic tuft cells are a newly defined terminally differentiated mTECs regulated by *Pou2f3*, which are similar to the molecular characteristics of the gut tuft cells (10, 11). It has been reported that thymic tuft cells express MHCII at a lower level than mTEC^hi^ (9). In line with this report, our RNA-seq analysis showed that tuft cell-associated gene set mainly expressed in mTEC^lo^ (**Figures 3A, B**). We found that the deletion of *Dhx9* in mTEC^lo^ led to a general decreased tuft cells-associated gene set, and the downregulated representative genes included *Lrmp, Avil, Trpm5, Dclk1, L1cam, Il25*, and more (**Figure 3A**), among which *Il25* played an important role in regulating the development of iNKT2 in the thymus (10, 49). We also noticed that mTEC^lo^ in *Dhx9* cKO mice exhibited a decreased TPM (transcripts per million) value of *Pou2f3* in RNA-seq data (**Figure 3C**). In addition, the genes involved in the taste transduction pathway were strikingly downregulated in mTEC^lo^ from *Dhx9* cKO compared with WT littermates (**Figure 3D**). Based on this relevant analysis, we further investigated the effect on the development of thymic tuft cells when Dhx9 was specifically knocked out in TECs. The percentage of DCLK1^+^ thymic tuft cells exerted a significant reduction in *Dhx9* cKO mice, as expected (**Figure 3E**). It is known that thymic tuft cells are essential for iNKT cell development (50). We analyzed CD1d-restricted iNKTs and its sub-lineages, distinguished by expression PLZF and RORγt. We found that the percentage of CD1d-restricted iNKT cells was significantly decreased in *Dhx9* cKO mice compared with WT littermates, leading to more dramatically decreased cell numbers (**Figures 3F, G**). Consistent with earlier studies in *Pou2f*3^-/-^mice (9, 10), further analysis showed reduction frequency and cell numbers in iNKT2 and iNKT17 in the thymus from *Dhx9* cKO mice, whereas iNKT1 merely displayed a decreased cell number (**Figures 3H, I**). Furthermore, it was recently reported that CD104^+^CCL21^+^ mTEC^lo^ were important for the development of iNKTs, especially iNKT1 and iNKT17, in the thymus *via* IL15 (49). The RNA-seq data showed that the expressions of *Ccl21a*, *CD104*, and *Il15* in mTEC^lo^ were comparable between WT and *Dhx9* cKO mice (**Figure S5**), which indicated that Dhx9 deficiency in TECs might not significantly influence the development of CD104^+^CCL21^+^ mTEC^lo^. In conclusion, Dhx9 was indispensable for the differentiation of thymic tuft cells by regulating the expression of *Pou2f3* and then the impaired thymic tuft cell differentiation in *Dhx9* cKO mice led to the retardation of iNKT cell development.

### The Homeostasis of Peripheral T Cell Pool Was Perturbed in *Dhx9* cKO Mice

**Figure 4:**
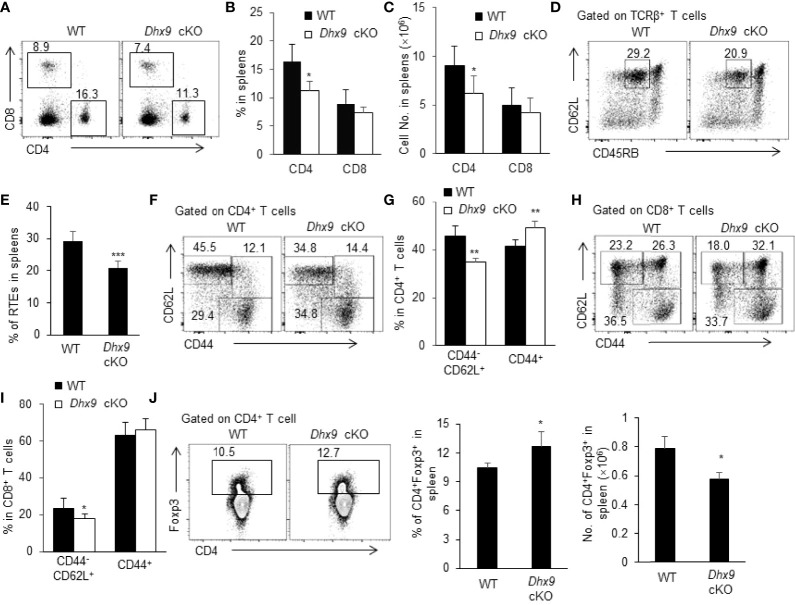
The homeostasis of periphery T cells was broken in *Dhx9* cKO mice. **(A–C)** The representative flow cytometry plots **(A)**, frequencies **(B)**, and numbers **(C)** of CD4^+^ T cell and CD8^+^ T cell from 4-week-old WT and *Dhx9* cKO mice. **(D, E)** The representative flow cytometry plots **(D)** and frequencies **(E)** of CD3^+^CD4^+^CD62L^+^CD45RB^int^ RTEs from 4-week-old WT and *Dhx9* cKO mice. **(F, G)** The representative flow cytometry plots **(F)** and frequencies **(G)** of CD62L^+^CD44^-^ naïve and CD44^+^ activated T cells in CD4^+^ T cells in 4-week-old WT and *Dhx9* cKO mice. **(H, I)** The representative flow cytometry plots **(H)** and frequencies **(I)** of CD62L^+^CD44^-^ naïve and CD44^+^ activated T cells in CD8^+^ T cells in 4-week-old WT and *Dhx9* cKO mice. **(J)** The representative flow cytometry plots, frequencies, and numbers of Treg cells in the spleen of indicated genotype mice. One representative histogram represented the mean ± SD for *n* = 5 mice in each group. The unpaired, two-tailed student’s *t*-test was used. *P < 0.05, **P < 0.01, ***P < 0.001 compared with WT control mice.

Mature SP thymocytes migrate from the thymus across the thymic blood endothelium to the peripheral T cell pool, where naive T cells retain cell number and diverse repertoire *via* antigen-independent homeostatic proliferation (51). We next investigated whether the severe thymus atrophy in *Dhx9* cKO mice influenced peripheral T cell homeostasis. The percentage and cell number of CD4^+^ and CD8^+^ lymphocytes were significantly reduced in the spleens of 4-week-old *Dhx9* cKO mice compared with WT littermates (**Figures 4A–C**). The decreased T cells in spleens can be partially caused by the reduced percentage of the recent thymic emigrants (RTEs), defined as CD4^+^CD62L^hi^CD45RB^int^ splenic lymphocytes (**Figures 4D, E**). Furthermore, according to the expression of CD44, we compared the activated state of T cells between WT and *Dhx9* cKO mice. We found that *Dhx9* cKO mice showed a significantly decreased percentage of CD44^-^CD62L^+^ naïve CD4^+^ and CD8^+^ T cells, whereas the percentage of CD44^+^ activation phenotype in both CD4^+^ and CD8^+^ T cells increased in *Dhx9* cKO mice (**Figures 4F–I**). Treg cells showed an increased percentage in *Dhx9* cKO mice, but the cell number decreased significantly (**Figure 4J**). Based on these data, we concluded that Dhx9 deficiency in TECs disturbed the homeostasis of peripheral T cell pool.

### Mice With a TEC-Specific Dhx9 Deletion Suffered From Autoimmune Disease

**Figure 5:**
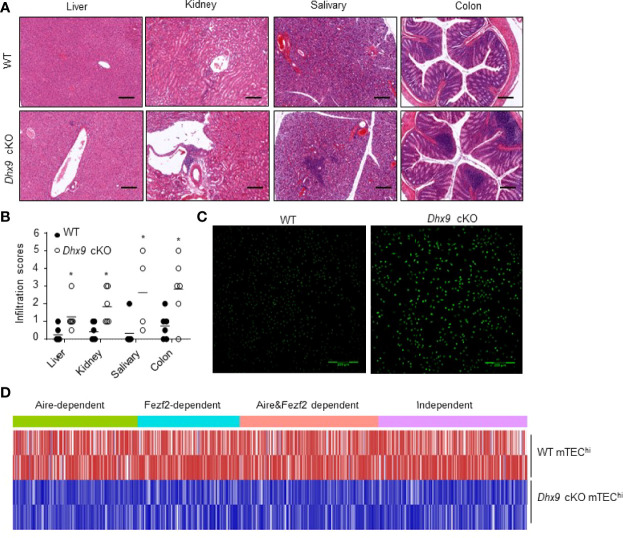
Mice with a TEC-specific Dhx9 deletion developed severe autoimmune turbulence. **(A)** Representative H&E stained histological sections of the liver, kidney, salivary, and colon from 8-month-old WT and *Dhx9* cKO mice, Scale bars: 200 μm. **(B)** Infiltration scores and means of H&E staining of WT and *Dhx9* cKO mice. One representative histogram represented the mean for *n* = 6 mice in each group. The unpaired, two-tailed student’s *t*-test was used. *P < 0.05, compared with WT control mice. **(C)** Antinuclear antibodies in the sera of 8-month-old WT and *Dhx9* cKO mice were examined using immunofluorescence staining in Hep-2 cell, Scale bars: 200 μm. **(D)** Heatmap of the downregulated TRA genes (*p* < 0.05) in mTEC^hi^ of WT and *Dhx9* cKO mice.

Severe atrophic thymus and disrupted T cell homeostasis in *Dhx9* cKO mice reminded us to observe whether *Dhx9* cKO mice developed an autoimmune phenotype spontaneously. As an important indicator of autoimmune disease, lymphocytic infiltration in multiple organs from 8-month-old WT, and *Dhx9* cKO mice were measured by hematoxylin and eosin (H&E) straining. The conjecture was confirmed by our observation that lymphocytic infiltration in the liver, kidney, salivary, and colon from *Dhx9* cKO mice were more obvious than in WT littermates (**Figure 5A**). Furthermore, according to the published lymphocytic infiltration scoring criteria, we found the majority of tissue in *Dhx9* cKO mice possessed a higher inflammatory infiltration score (**Figure 5B**). We also compared the contents of antinuclear antibodies in sera from WT littermates and *Dhx9* cKO mice. Brighter green fluorescence in *Dhx9* cKO indicated high levels of antinuclear antibodies in the sera of *Dhx9* cKO mice (**Figure 5C**).

It is known that the mature mTECs function as special antigen-presenting cells to eliminate self-reactive T cells by expressing a range of TRA profiles (52, 53). The expression of TRAs is mainly controlled by Aire and Fezf2 (54, 55). Our RNA-seq analyses showed that both Aire and Fezf2-dependent or independent TRAs were affected after *Dhx9* deletion in TECs (**Figure 5D**). Compared with WT, the RNA-seq data revealed that most TRAs were downregulated significantly in *Dhx9* cKO mice (**Figure 5D**). These results suggested that Dhx9 deletion in TECs obviously decreased TRA expression, so it influenced the immune tolerance establishment and finally caused the spontaneous occurrence of autoimmune diseases.

### Dhx9 Deficiency Impaired mTEC Proliferation and Cell Cycle Progression

**Figure 6:**
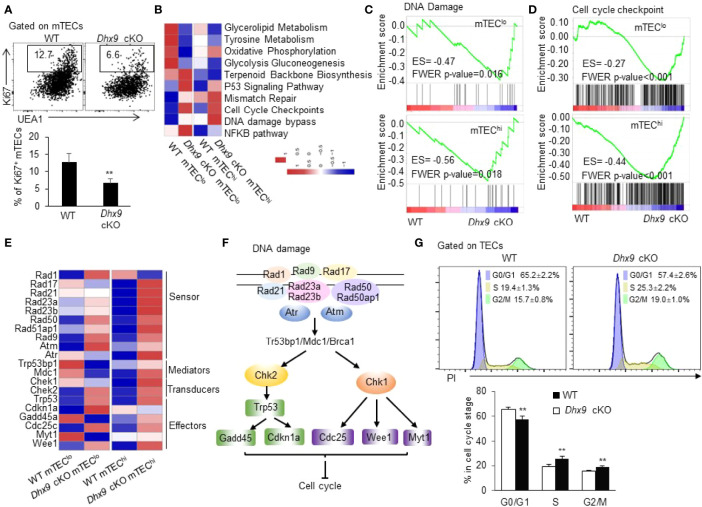
Dhx9 ablation in TECs impaired cell proliferation *via* cell cycle arrest. **(A)** Representative flow cytometric plots and frequency of cells intracellularly stained with Ki67 in mTECs from 4-week-old WT and *Dhx9* cKO mice (*n* = 5 for each group). **P < 0.01 (student’s *t*-test). **(B)** A row-normalized heatmap showing the changed cellular metabolism and proliferation-related gene pathways in mTEC^lo^ and mTEC^hi^ from WT and *Dhx9* cKO mice analyzed by GSVA. The cutoff criterion was *P* < 0.05. **(C)** GSEA exhibited an enrichment of the DNA damage gene set in *Dhx9*-deficient mTEC^lo^ and mTEC^hi^, as defined by the criterion *P* < 0.05. **(D)** An enrichment of the cell cycle checkpoint gene set in Dhx9-deficient mTEC^lo^ and mTEC^hi^ analyzed by GSEA (*P* < 0.05). **(E)** Heatmap of the significant changed genes (*P* < 0.05) associated with the DNA damage in mTEC^lo^ and mTEC^hi^ from WT and *Dhx9* cKO mice. **(F)** The molecular network of gens listed in **(E)** describing the connection between the DNA damage process and cell cycle arrest. **(G)** The cell cycle distribution for WT and *Dhx9* KO TECs (*n* = 4 for each group) was measured using propidium iodide (PI) straining *via* flow cytometry. The data were shown as mean ± SD. **P < 0.01 (student’s *t*-test).

Given the significant reduced cell number of mTECs in *Dhx9* cKO mice, we wondered whether Dhx9 inactivation impaired the survival and proliferation ability of mTECs in the adult thymus. To examine this hypothesis, we analyzed TECs from 4-week-old WT and *Dhx9* cKO mice with intracellular staining with antibodies against the proliferation marker Ki67 and the apoptosis indicator active caspase 3 (**Figures 6A** and **S6**). The results showed a significant reduced percentage of Ki67^+^ mTECs in *Dhx9* cKO mice compared with WT littermates (**Figure 6A**), whereas there was no difference in caspase 3^+^ apoptotic mTECs and the expression of cell death-associated genes between WT and *Dhx9* cKO mice (**Figures S6A, B**). Therefore, Dhx9 deficiency mainly impaired the maintenance of mTEC proliferation in mice.

To further investigate the regulation of Dhx9 in mTEC proliferation, we performed a RNA-seq analysis of mTEC^lo^ and mTEC^hi^ of WT and *Dhx9* cKO mice (**Figure S2**). In consideration of Dhx9 basic biological function, we noticed that the DNA damage signaling pathway was significantly upregulated in both mTEC^lo^ and mTEC^hi^ from *Dhx9* cKO mice, accompanied by upregulated cell cycle checkpoint pathways (**Figures 6B–D**). The extensive upregulated expression of many DNA damage-associated genes in mTEC^hi^ of *Dhx9* cKO mice was observed, compared with WT littermates (**Figure 6E**), which were involved in the overall process of DNA damage process, as illustrated in **Figure 6F**. It has been well demonstrated that DNA damage can be detected by damage sensor proteins, such as ATM, ATR, and the Rad17-RFC complex. Additionally, downstream Chk1 and Chk2 Ser/Thr kinases initiate signal transduction cascades, which induce cell cycle arrest during G_1_ to S, DNA replication, or G_2_ to mitosis phase (56). The upregulated p53 signaling pathway in *Dhx9* cKO mice transcriptionally induced the expression of the cyclin-dependent kinase inhibitor cdkn1a, which facilitated DNA repair by cell-cycle exit at the G1 phase and also participated in G_2_/M cell cycle arrest (57–59) (**Figures 6B, E**). Moreover, cell cycle regulating kinases Wee1 and Myt1, responsible for inhibitory Cdk1 phosphorylation during DNA damage-caused G_2_ cell cycle arrest (60, 61), were also upregulated in *Dhx9* cKO mice compared with WT littermates (**Figure 6E**). Modulated expressions of these genes likely contributed to the poor mTEC proliferation in *Dhx9* cKO mice. The effect of *Dhx9* deficiency on cell-cycle distribution was examined by flow cytometric analysis with propidium iodide (PI) staining. The results showed the decreased G0/G1 phase and accumulated S and G2/M phases in *Dhx9*-deleted TECs compared with WT TECs. These results suggested that Dhx9 deficiency led to cell cycle arrest, mostly at the S and G2/M phases (**Figure 6G**).

The related studies indicated that cell cycle regulators had a crosstalk with glycolysis (62, 63). It was reported that cyclin D1 could negatively regulate the hexokinase II abundance (64). Xiao et al. found that glucose transporter 1 (GLUT1) acted as a link between glycolysis and proliferation during the progression of prostate cancer (65). The gene set variation analysis (GSVA) showed an extensive downregulation of glycometabolism-related signaling pathways in the mTEC^lo^ and mTEC^hi^ of *Dhx9* cKO mice compared with corresponding WT TECs (**Figures 6B** and **S7**, **S8**). It were proved that TECs proliferated extensively, with a high turnover rate during thymus growth (66). Cell proliferation requires many substrates, such as elevated metabolic intermediates (e.g., glucose-6-phosphate, acetyl-CoA, and fructose-6-phosphate) and continued regeneration of cofactors to provide free energy or reduce equivalents for reactions (e.g., ATP, NADPH, and NADH) (67). The retarded glycolysis and oxidative phosphorylation in *Dhx9*-deleted mTECs might contribute to the poor TEC proliferation.

### Dhx9-Deficient mTECs Overactivated NF-κB Signaling Pathway

**Figure 7:**
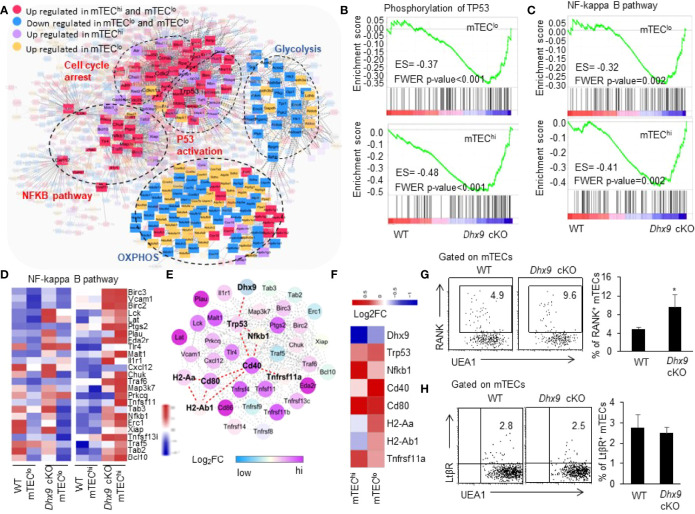
TEC-specific inactivation of Dhx9 leads to over-activation of NF-κB signaling pathways in a P53-dependent manner. **(A)** The molecular network associated with P53. **(B)** GSEA exhibited an enrichment of the phosphorylation of the TP53 gene set in *Dhx9*-deficient mTEC^lo^ and mTEC^hi^, as defined by the criterion *P* < 0.05. **(C)** GSEA exhibited an enrichment of the NF-κB signaling pathway in Dhx9-deficient mTEC^lo^ and mTEC^hi^, as defined by the criterion *P* < 0.05. **(D)** Heatmap of the significantly changed genes (*p* < 0.05) associated with the NF-κB signaling pathway in mTEC^lo^ and mTEC^hi^ from WT and *Dhx9* cKO mice. **(E)** The molecular network between the P53 and NF-κB pathway associated with upregulated genes. **(F)** The heatmap showed log_2_FC of indicated genes in *Dhx9* deficiency in mTEC^lo^ and mTEC^hi^, compared with the WT control. **(G)** Representative flow cytometric plots and frequencies of RANK^+^ mTECs from 4-week-old WT (*n* = 4) and *Dhx9* cKO mice (*n* = 5). *P < 0.05, (student’s *t*-test). **(H)** Representative flow cytometric plots and frequencies of LtβR^+^ mTECs from 4-week-old WT and *Dhx9* cKO mice (*n* = 4 for each group). *P < 0.05, (student’s *t*-test).

As illustrated in **Figure 1**, we noticed significant accelerated mTEC maturation kinetics in *Dhx9* cKO mice. To determine the mechanisms that facilitated this maturation process, we performed a network analysis based on the differential expressed genes between WT and *Dhx9* cKO mTECs. The network uncovered the central regulatory role of the p53 signaling pathway, and the enrichment of p53 phosphorylation-related genes in both mTEC^lo^ and mTEC^hi^ from *Dhx9* cKO mice increased the stability of P53 protein through posttranslational modifications (**Figures 7A, B**) (68, 69). Except for upregulation of cell cycle arrest, the network analysis showed genes included in glycolysis and OXPHOS were significantly downregulated in *Dhx9*-deficient mTECs, and it was consistent with the existing opinion that p53 negatively regulated cellular metabolism (**Figures 6B**, **7A**) (70, 71). The upregulated genes involved in the NF-κB pathway in *Dhx9* cKO mice caught our attention (**Figure 7A**). It is well demonstrated that the activation of the NF-κB pathway plays an important role in the maturation of mTECs, guiding us to focus on the NF-κB pathway in *Dhx9*-inactivated mTECs. GSEA revealed that the NF-κB pathway was enriched in both mTEC^lo^ and mTEC^hi^ of *Dhx9* cKO mice (**Figure 7C**). The expressions of many NF-κB pathway-related genes increased in *Dhx9*-deficient mTEC^hi^ and to a lesser degree in *Dhx9*-deficient mTEC^lo^ (**Figure 7D**). Subsequently, we wondered how p53 regulated the NF-κB pathway in *Dhx9*-deleted mTECs. The associated gene network reminded us that the regulation might indirectly upregulate the TNFRSF (**Figure 7E**). Previous studies showed that p53 promoted the expression of Tnfrsf11a (RANK) at the transcriptional level (18). We found the expression of CD40 and Tnfrsf11a increased notably in *Dhx9*-deficient mTECs in comparison with the WT control (**Figure 7F**). We next evaluated the expression of RANK in mTECs from WT and *Dhx9* cKO mice *via* flow cytometry. Consistent with the upregulation of CD40 (**Figure 1H**), the percentage of RANK^+^ mTECs were increased significantly in 4-week-old *Dhx9* cKO mice (**Figure 7G**). However, the expression of LtβR had no significant difference between WT and *Dhx9* cKO TECs (**Figure 7H**). These data collectively indicated that Dhx9 deficiency activated the positive regulatory loop of the activated NF-κB pathway and enhanced the expression of CD40 and RANK in mTECs.

## Discussion

As the most important components of the thymic microenvironment, TECs support the development of thymocytes and govern the TCR repertoires selection for efficient establishment of T cell self-tolerance and immunity (5). In this study, we identified Dhx9 as a crucial determinant for the normal development and maturation of mTECs. Conditional ablation of *Dhx9* in TECs caused a significant thymus hypoplasia, manifested by atrophic thymus, especially a shrunken medulla region. Further analysis showed that *Dhx9* specifically deleted in TECs possessed lower percentage and cell number of competent mTECs compared with WT littermates, including different mature stages of mTECs identified with the expressions of CD80, MHCII, and Aire, as well as the newly identified thymic tuft cells. However, the cell number of cTECs was similar in WT and *Dhx9* cKO mice. With the deeper insight into specific mechanisms by bioinformatic and flow cytometry assays, we concluded that Dhx9 inactivation mainly damaged the proliferation of mTECs instead of cell death. Importantly, incompetent mTECs in *Dhx9* cKO mice impaired T cell differentiation, inhibited the central immune tolerance establishment, and caused autoimmune disorders in mice.

TECs exhibited extensive proliferation during the thymic growth period, and about 10% of newly amplified TECs were produced each day (66). In *Dhx9* cKO mice, the significant reduced percentage of Ki67^+^ mTECs suggested an impaired proliferation of *Dhx9*-deleted mTECs. It was reported that Dhx9 had the ability to maintain the stabilization of the genome by unwinding complex nucleic acid structures, which were formed as transient intermediates during DNA replication and recombination (22). Dhx9 interacted with several DNA damage response-associated proteins, such as BRCA1 and Ku86 (72, 73). Moreover, Dhx9 inactivation could promote the accumulation of R-loop and induced DNA damage (74). *In vitro* assays, it was shown that Dhx9 could resolve an aberrant structure, H-DNA, to confine genomic instability (75). Based on the relevant research, NTP-dependent Dhx9 helicase activity is important for maintaining genome stability. With the help of RNA-seq analysis, we found the DNA damage response was upregulated significantly in mTECs after *Dhx9* deletion. As reported, DNA damage elicits a cell cycle arrest that allows time for activating repair pathways to ensure subsequent phases of the cell cycle (76). Inactivation of *Dhx9* in TECs showed an upregulation of cell cycle checkpoint genes. We concluded that *Dhx9* deficiency in TECs destroyed genome stability, caused a DNA damage response, and subsequently induced cell cycle arrest, as indicated by upregulation of the cell cycle checkpoint, which impaired the proliferation of *Dhx9*-deleted mTECs.

The upregulation of CD40, CD80, MHCII, and Aire expressions in mTECs is important for mTEC differentiation and functional maturation, and the NF-κB signaling pathway plays an important role in this process (32, 77). Our RNA-seq analysis revealed the NF-κB signaling pathway was remarkably upregulated in *Dhx9*-deficient mTECs. Further analysis showed the percentage of mature mTECs highly expressing CD80, MHCII, and Aire were significantly increased in *Dhx9*-deleted mTECs. It was reported that Dhx9 positively regulated the NF-κB signaling pathway during the innate immune response, serving as a bridging factor to recruit NF-κB p65 and RNA polymerase II (RNAPII) to the NF-κB-specific promoters, or acting as the cytosolic DNA-sensor (21, 22, 78). Dhx9 might not function as a direct mediator in the regulation of the NF-κB signaling pathway in TECs, as indicated by studies on the roles of Dhx9 in innate immune response (21, 22, 78). Additionally, TNFRSF results in the activation of the NF-κB signaling pathway *via* the interaction between RANK, CD40, and LtβR and their corresponding ligand (37). Our results showed an elevated expression of CD40 and RANK but not LtβR in *Dhx9* cKO mice. Previous studies showed that the transcription start site of *Tnfrsf11a* (RANK) contained p53 response elements and p53 regulated RANK expression to influence the maturation of mTECs (18). Thus, based on the over-expression of p53 in *Dhx9*-deleted mTECs, we presumed the upregulated p53-RANK-NF-κB signaling pathway axis contributed to the accelerated mTEC maturation in *Dhx9* cKO mice. It would be more convinced with additional experiments using mice with Dhx9 and p53 double deficiency to address this issue, which needed to be studied in the future.

Over the past several years, mTECs have been identified as a cell population with a high degree of internal heterogeneity (7). Our results showed that loss of *Dhx9* blocked the differentiation of thymic tuft cells, a terminally differentiated mTECs characterized by the canonical taste transduction pathway (9, 10). Our RNA-seq analysis displayed a significant downregulated tuft cells-associated gene set and the taste transduction pathway in *Dhx9* cKO mice. Furthermore, as the critical regulator of thymic tuft cell differentiation, the transcription factor *Pou2f3* was downregulated in the *Dhx9*-deleted mTEC^lo^ population. The decreased *Pou2f3* expression might cause a blockage of thymic tuft cell differentiation in the *Dhx9* deleted thymus. Consistently, the percentage of DCLK1^+^ thymic tuft cells reduced significantly in *Dhx9* cKO mice, and its function was also impaired, as indicated by the blocked development of iNKT cells, especially iNKT2 cells. On the other hand, Lucas et al. illustrated that CD104^+^CCL21^+^ mTEC^lo^ also regulated the development of iNKT1 and iNKT17 cell in the thymus *via* IL15 (49, 79). However, there was no significant reduced *CCl21a* and *il15* expression in the RNA-seq data of *Dhx9* cKO mTEC^lo^ compared with WT mTEC^lo^. Based on the iNKT cells development deficiency in *pou2f3* ^-/-^ mice (49, 79), we speculated that blocked iNKT cells development in *Dhx9* cKO mice might be mainly caused by the impaired thymic tuft cells. Although the potential involvement of CD104^+^CCL21^+^ mTEC^lo^ in the poor iNKT cells development in *Dhx9* cKO mice was unlikely as evidenced by the present data, the detailed investigation on CD104^+^CCL21^+^ mTEC^lo^ development in *Dhx*9 cKO mice was still required.

Competent mTECs activate self-tolerance by stochastic encounters between self-reactive T cells and mature mTECs or dendritic cells presenting TRAs from mTECs (80). The disruption of the mTEC differentiation and maturation in *Dhx9* cKO mice led to a failure of thymopoiesis, including thymocyte selection, CD4SP and CD8SP post-selection maturation, and nTreg development. The peripheral T cell pool has two cellular compartments, including naive T cells, mainly supplied by RTEs and self-renewing activated/memory T cells (81, 82). *Dhx9* cKO mice showed reduced cell numbers of RTEs, naïve T cells and Treg cells, and an increased level of activated/memory T cells. Despite the accelerated maturation of mTECs in *Dhx9* cKO mice, the RNA-seq analysis revealed that *Dhx9* ablation remarkably interfered in the expression of many TRAs in mTECs, which were important for self-tolerance establishment (83). Consistent with regarding defects T cell development and poor TRA expression in mTECs in *Dhx9* cKO mice, Dhx9 deficiency in TECs resulted in spontaneous autoimmune diseases, characterized by obvious organ lymphocyte infiltration and the existence of antinuclear antibodies. Hence, Dhx9 expressed in TECs was indispensable for the establishment of T cell central tolerance.

In a summary, our study showed that Dhx9 was an important regulation for the development and maturation of mTECs and was indispensable for the central immune tolerance establishment and the prevention of autoimmune diseases. As illustrated in **Figure S9**, the inactivated Dhx9 in TECs caused a DNA damage response, impaired the proliferation of mTECs by upregulating cell cycle arrest, and subsequently decreased cell number of mTECs and thymic tuft cells. The over-expressed p53 and its related pathways in Dhx9-deficient mTECs accelerated the maturation kinetics of mTECs *via* the RANK/CD40-NF-κB axis. Our research suggested Dhx9 in TECs functioned as a novel guardian of thymus development and function.

## Data Availability Statement

The datasets presented in this study can be found in online repositories. The names of the repository/repositories and accession number(s) can be found below: https://ngdc.cncb.ac.cn/, PRJCA008466.

## Ethics Statement

The animal study was reviewed and approved by the Animal Ethics Committee of the Institute of Zoology (Beijing, China). Written informed consent was obtained from the owners for the participation of their animals in this study.

## Author Contributions

Designed and performed the experiments: XD and QZ. The bioinformatic analysis of RNA-seq: JZ. Writing-original draft: XD and ZL. Methodology: QZ and YX. Created knockout mice: BZ. Supervision: BZ and YZ. All authors contributed to the article and approved the submitted version.

## Funding

This work was supported by grants from the National Natural Science Foundation for Key Program (31930041, YZ), the National Key Research and Development Program of China (2017YFA0105002, 2017YFA0104401, and 2017YFA0104402, YZ), and the Knowledge Innovation Program of the Chinese Academy of Sciences (XDA16030301, YZ).

## Conflict of Interest

The authors declare that the research was conducted in the absence of any commercial or financial relationships that could be construed as a potential conflict of interest.

## Publisher’s Note

All claims expressed in this article are solely those of the authors and do not necessarily represent those of their affiliated organizations, or those of the publisher, the editors and the reviewers. Any product that may be evaluated in this article, or claim that may be made by its manufacturer, is not guaranteed or endorsed by the publisher.

## Abbreviations

Aire, Autoimmune Regulator; CD4SP, CD4 Single-Positive Thymocytes; CD8SP, CD8 Single-Positive Thymocytes; cTECs, Cortical Thymic Epithelial Cells; DMEM, Dulbecco’s Modified Eagle’s Medium; DN, Double Negative; DP, Double Positive; FACS, Fluorescence-Activated Cell Sorting; FBS, Fetal Bovine Serum; Foxp3, Forkhead Box P3; GSVA, Gene Set Variation Analysis; H&E, Hematoxylin and Eosin Staining; iNKTs, Invariant Natural Killer T Cells; KEGG, Kyoto Encyclopedia of Genes and Genomes; LtβR, Lymphotoxin β Receptor; MFI, Mediate Fluorescence Intensity; MHCII, Major Histocompatibility Complex Class II; mTECs, Medullary Thymic Epithelial Cells; PI, Propidium Iodide; RANK, Receptor Activator for NFκB; RTEs, Recent Thymic Emigrants; SD, Standard Deviations; TECs, Thymic Epithelial Cells; TRAs, Tissue Restricted Antigens; Treg, Regulatory T Cell; UEA1, Ulex Europaeus Lectin 1.
