# Supplementary material for: Cytosolic Nuclear Sensor Dhx9 Controls Medullary Thymic Epithelial Cell Differentiation by p53-Mediated Pathways
Source: Front Immunol. 2022 Jun 3;13:896472. doi: 10.3389/fimmu.2022.896472 (PMC9203851; doi:10.3389/fimmu.2022.896472)
Supplement: Supplementary file 1 [file DataSheet_1.pdf]

Cytosolic nuclear sensor Dhx9 controls medullary thymic epithelial cell differentiation by p53-mediated pathways

Xue Dong[1,2,#], Jiayu Zhang[1,2,#], Qian Zhang[1,2,#], Zhanfeng Liang[1,2,3,#], Yanan Xu[1,2], Yong Zhao[1,2,3,*], Baojun Zhang[4,*]

[1] State Key Laboratory of Membrane Biology, Institute of Zoology, Chinese Academy of Sciences, Beijing. [2] University of Chinese Academy of Sciences, Beijing, China. [3] Beijing Institute for Stem Cell and Regeneration, Beijing, China. [4] Department of Pathogenic Microbiology and Immunology, School of Basic Medical Sciences, Xi'an Jiaotong University, Xi'an, Shaanxi, China.

Running head: Regulatory role of Dhx9 on thymus development

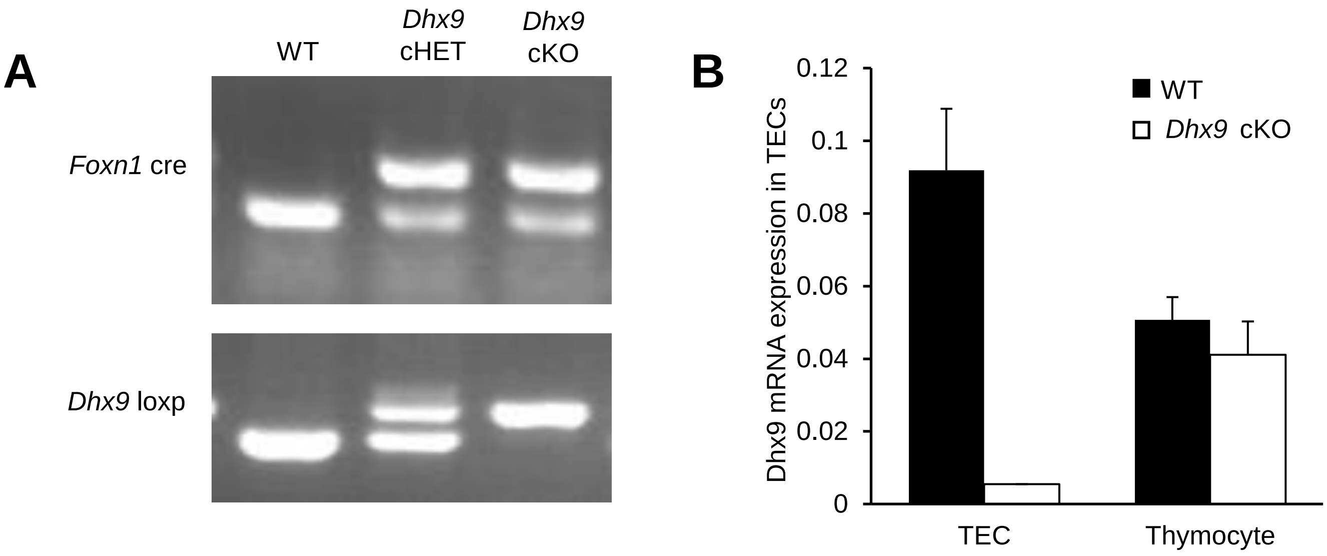

Figure S1. Establishment and identification of mice with a TEC-specific *Dhx9* deletion.
(**A**) DNA genotyping identification by agarose gel electrophoresis. (**B**) The mRNA expression of *Dhx9* in TECs and thymocytes from WT and *Dhx9* cKO mice

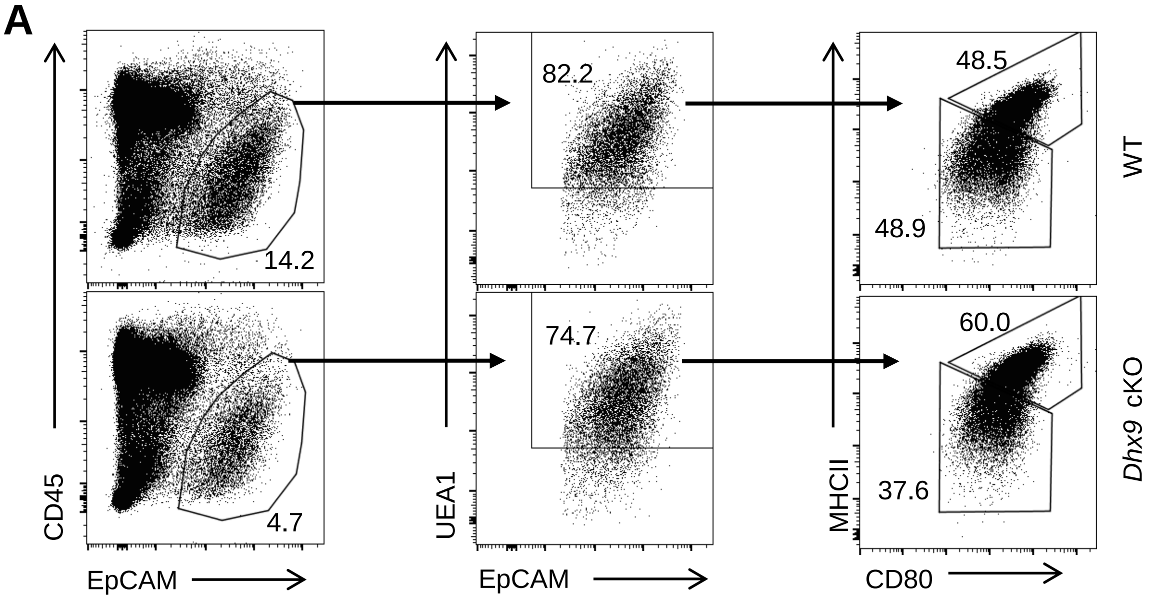

Figure S2. Sorting strategies for mTEC^{hi} (CD45^{-}EpCAM^{+}UEA1^{+}Ly51^{-}CD80^{+}MHCII^{hi}) and mTEC^{lo} (CD45^{-}EpCAM^{+}UEA1^{-}Ly51^{+}CD80^{-}MHCII^{lo}) from WT and *Dhx9* cKO mice.

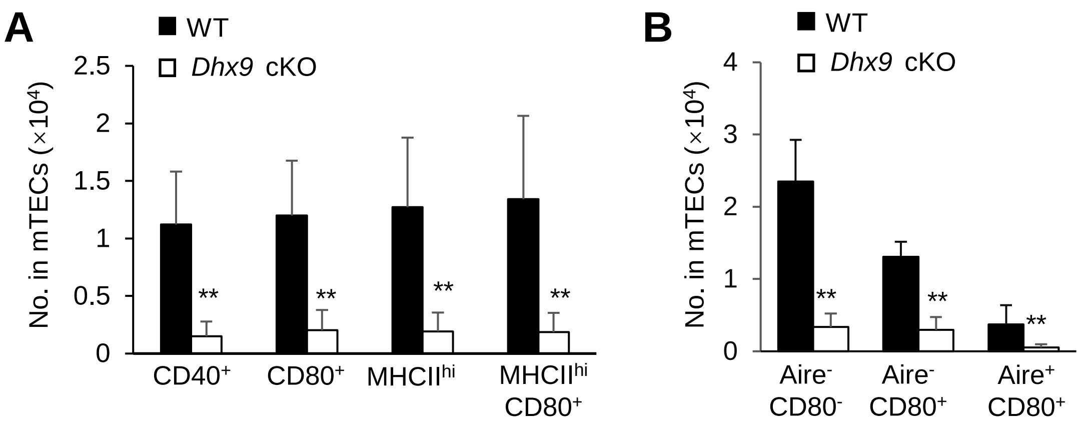

Figure S3. Cell number of mature mTECs expressing CD40, CD80, MHCII or Aire obviously decreased in *Dhx9* cKO mice.

(**A**) Cell number of the maturation of mTECs measured by the expression of CD40, CD80 and MHCII in 4-week-old WT (n=8) and *Dhx9* cKO mice (n=6). (**B**) Cell number of the mature stage by the expression of CD80 and Aire in 4-week-old WT (n=8) and *Dhx9* cKO mice (n=6). One representative histogram represented the mean ± SD. The unpaired, two-tailed student's t-test was used. *P < 0.05; **P < 0.01, ***P < 0.001 compared with WT control mice.

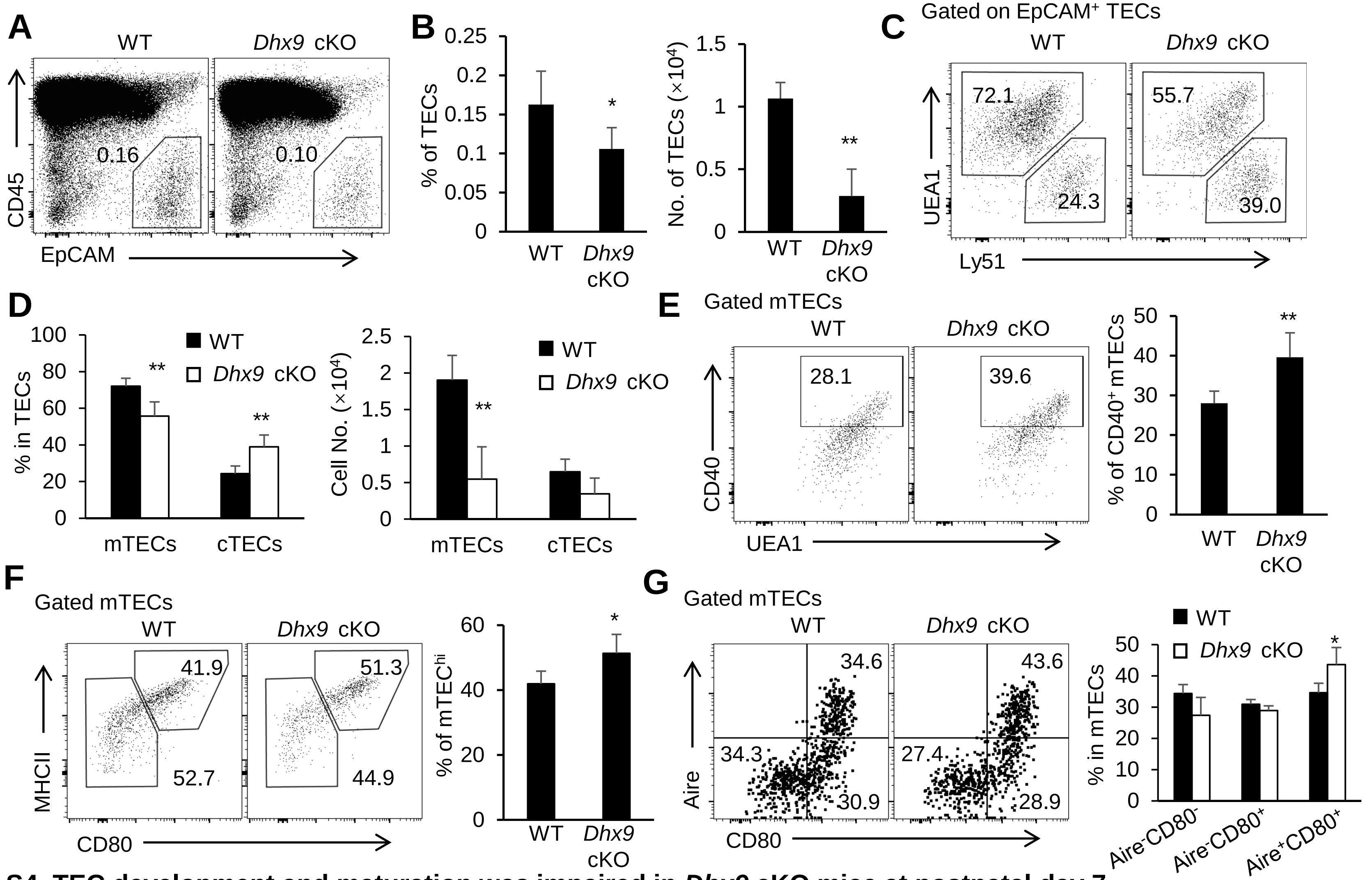

Figure S4. TEC development and maturation was impaired in *Dhx9* cKO mice at postnatal day 7.

(**A-B**) Flow cytometric profiles, frequencies, and absolute cell numbers of TECs in 1-week-old WT (n=4) and *Dhx9* cKO mice (n = 5). (**C-D**) Representative flow cytometry plots, frequencies, and cell numbers of mTECs and cTECs from 1-week-old WT and *Dhx9* cKO mice (n = 4 for each group). (**E**) Representative flow cytometry plots and frequencies of $CD40^+$ mTECs in 1-week-old WT and *Dhx9* cKO mice (n = 4 for each group). (**F**) Representative flow cytometry plots and frequencies showed the maturation of mTECs, as measured by the expression of CD80, and MHCII in 1-week-old WT and *Dhx9* cKO mice (n = 4 for each group). (**G**) Representative flow cytometry plots and frequency exhibited the mature stage by the expression of CD80 and Aire in 1-week-old WT (n = 4) and *Dhx9* cKO mice (n = 5). Data were shown as the mean ± SD. The unpaired, two-tailed student's t-test was used. $^*P < 0.05$, $^{**}P < 0.01$, $^{***}P < 0.001$ compared with WT control mice.

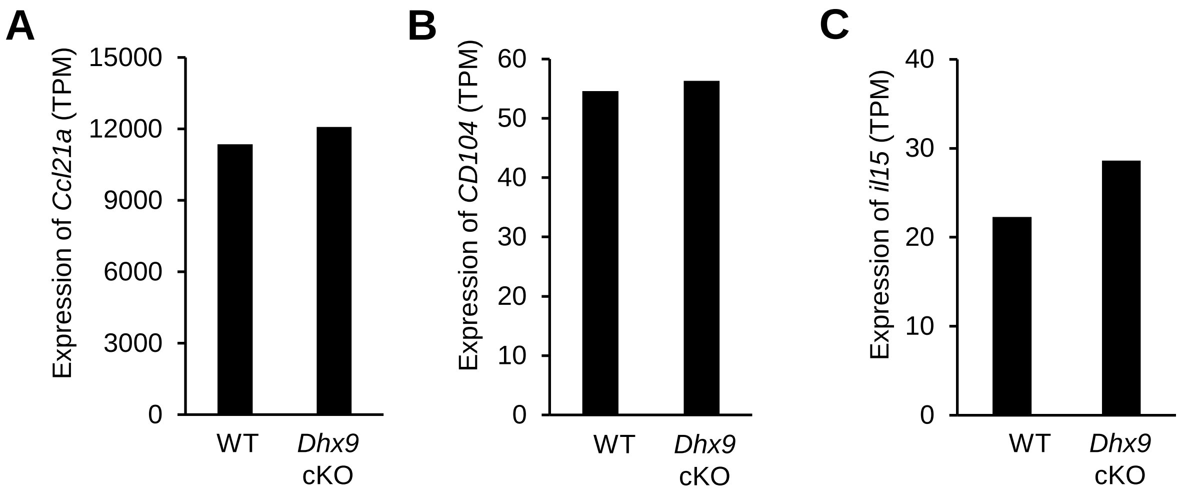

Figure S5. Dhx9 inactivation in TECs did not change the development of CD104^{+}CCL21^{+} mTEC^{lo}. (**A-C**) The TPM value of CD104^{+}CCL21^{+} mTEC^{lo} associated genes *Ccl21a* (**A**), *CD104* (**B**), and *il15* (**C**) in mTEC^{lo} of WT and *Dhx9* cKO mice according to the bulk RNA-seq data.

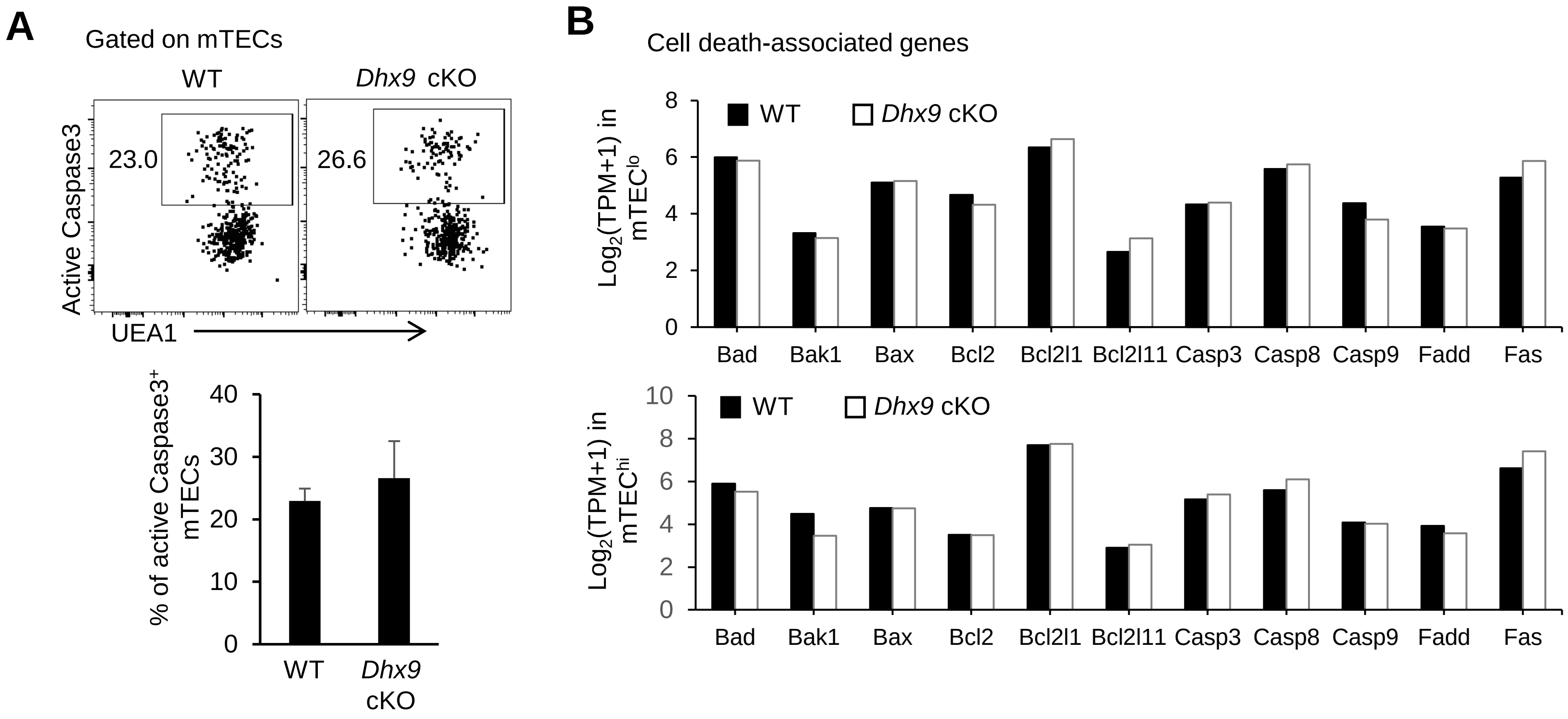

Figure S6. Dhx9 ablation had no effect on cell apoptosis of mTECs.
(**A**) Representative flow cytometric plots and frequency of active caspase3$^+$ mTECs from 4-week-old WT and *Dhx9* cKO mice (n=5 for each group). (**B**) The expression of cell death associated genes (TPM) in mTEC^{lo} and mTEC^{hi} of WT and *Dhx9* cKO mice according to the bulk RNA-seq data.

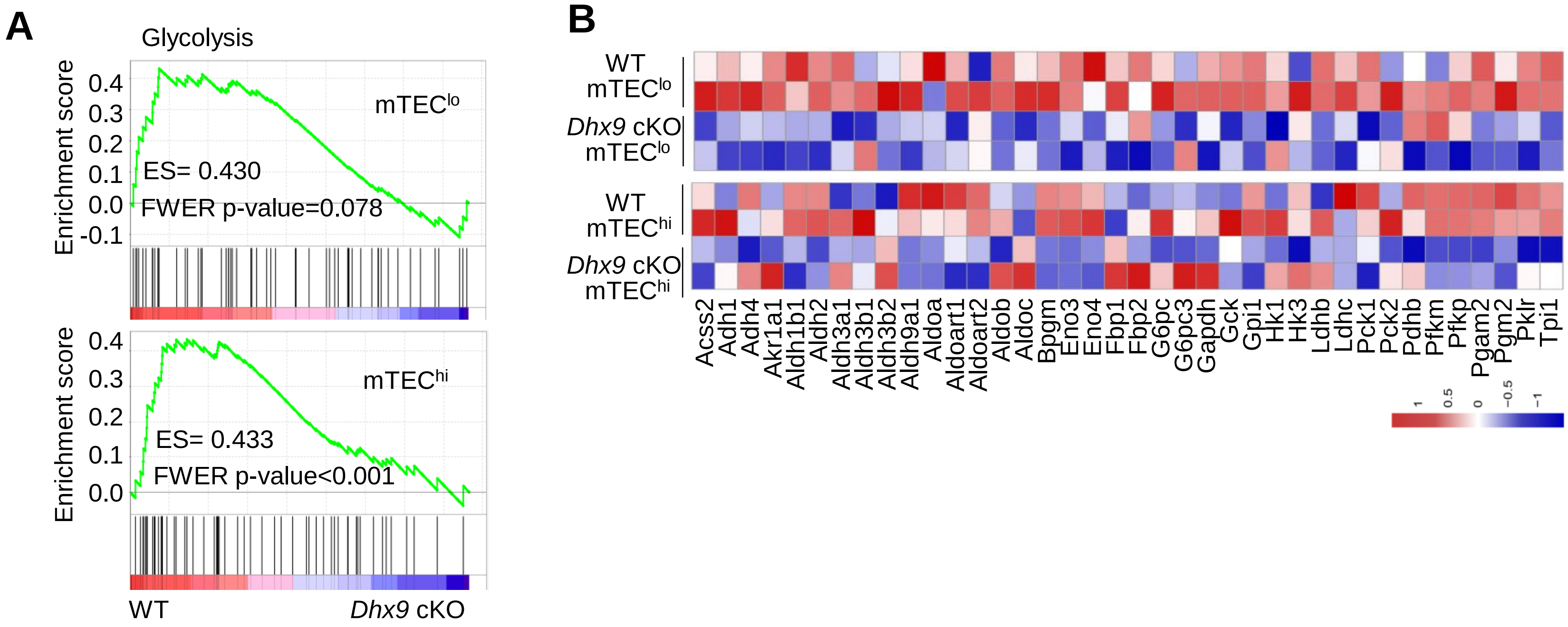

Figure S7. The glycolysis gene set was down-regulated in Dhx9-deficient mTEC^{lo} and mTEC^{hi}.
(**A**) GSEA was performed with the genes involved in the glycolysis pathway in mTEC^{lo} and mTEC^{hi} from WT and *Dhx9* cKO mice according to the RNA-seq data. (**B**) Heat map showing the expression of genes involved in the glycolysis pathway in mTEC^{lo} and mTEC^{hi} from WT and *Dhx9* cKO mice according to the RNA-seq data.

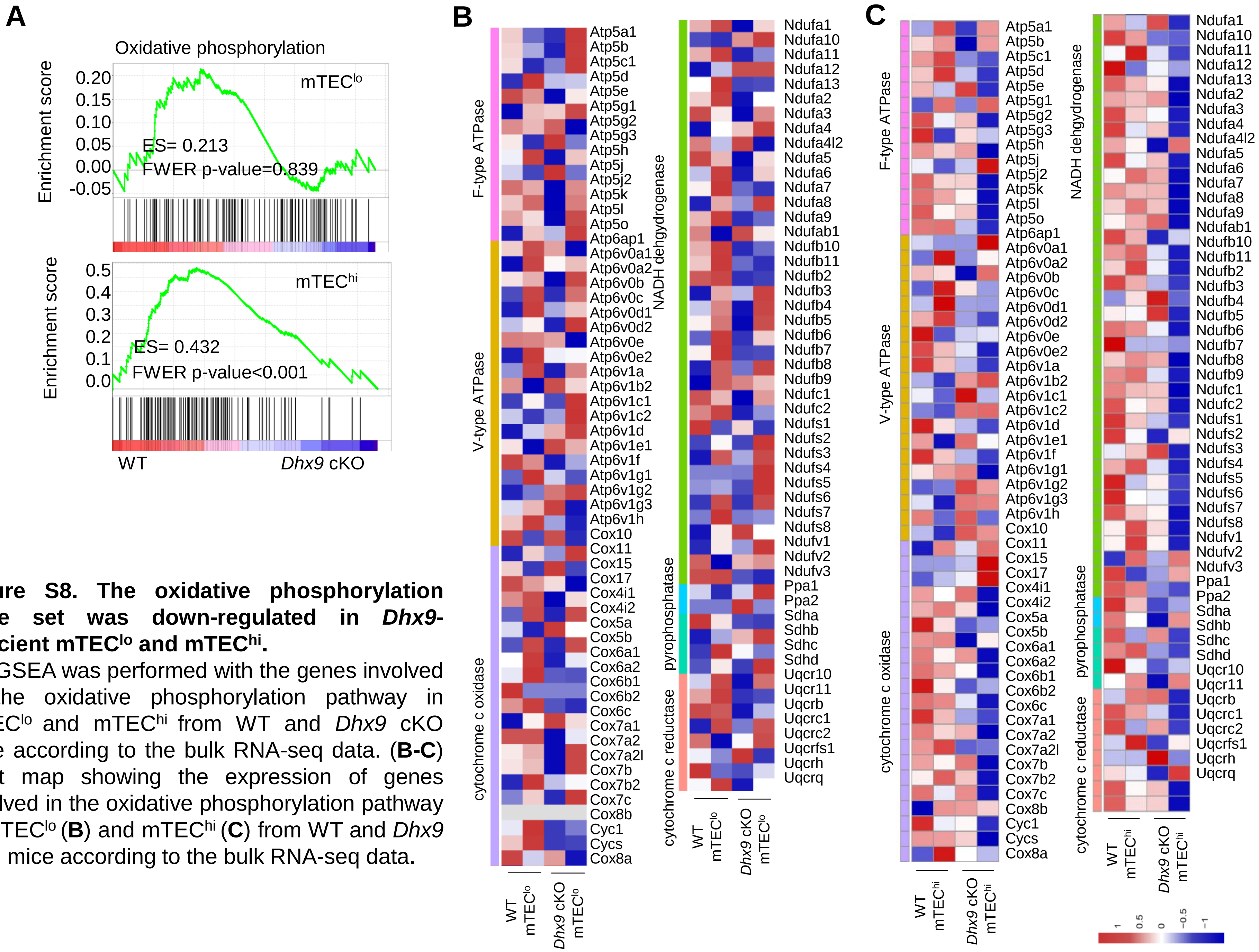

Figure S8. The oxidative phosphorylation gene set was down-regulated in *Dhx9*-deficient mTEC^{lo} and mTEC^{hi}.

(**A**) GSEA was performed with the genes involved in the oxidative phosphorylation pathway in mTEC^{lo} and mTEC^{hi} from WT and *Dhx9* cKO mice according to the bulk RNA-seq data. (**B-C**) Heat map showing the expression of genes involved in the oxidative phosphorylation pathway in mTEC^{lo} (**B**) and mTEC^{hi} (**C**) from WT and *Dhx9* cKO mice according to the bulk RNA-seq data.

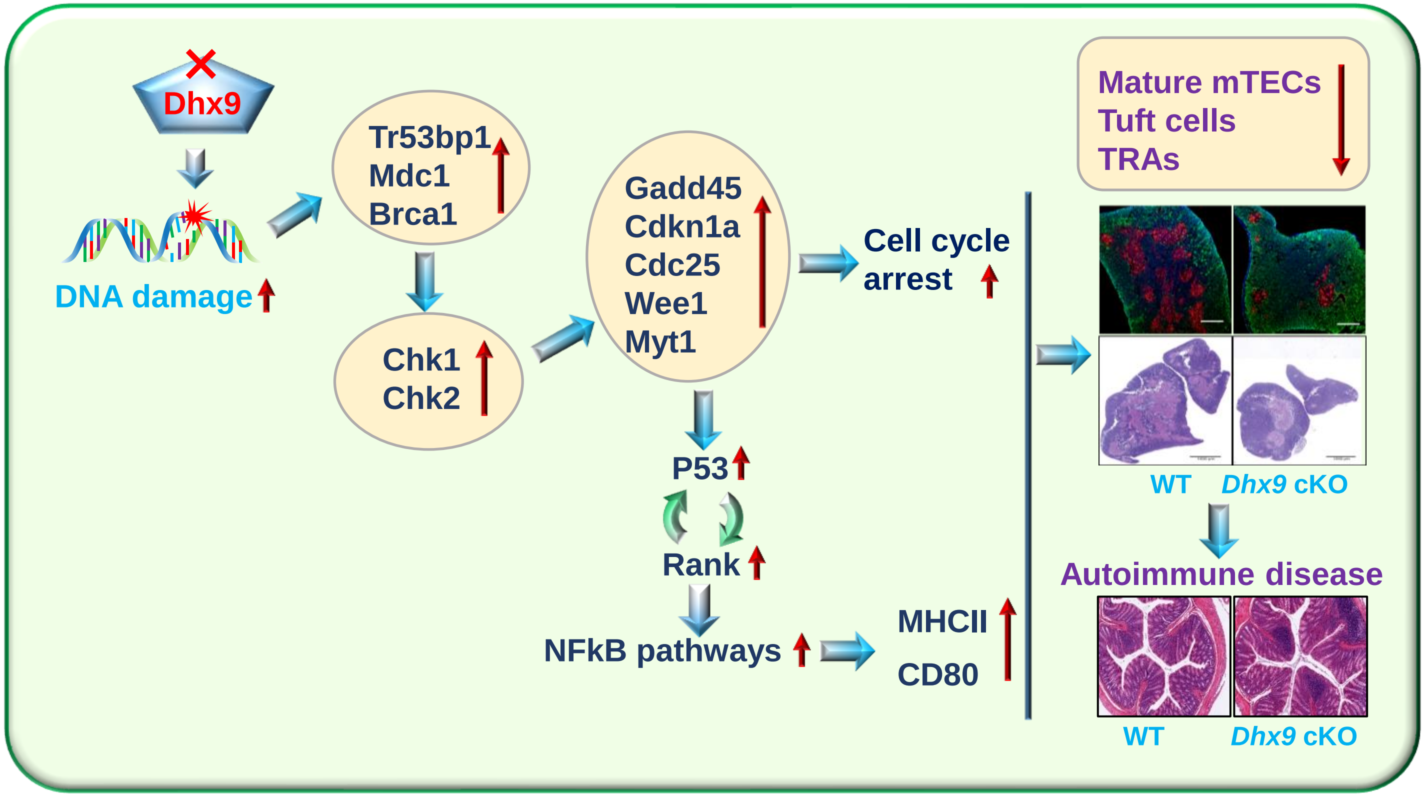

Figure S9. The visual abstract illustrate the regulate mechanism of Dhx9 in the development and maturation of TECs.

Dhx9 ablation in TECs induced the DNA damage response and the subsequently elevated cell cycle arrest and impaired the development of mTECs, embodied by a atrophic thymus with a significant reduced cell number of mTECs and thymic tuft cells. Simultaneously, as an important regulator of cell cycle arrest, the up-regulated P53 could promote the expression of RANK and accelerate the maturation of mTECs via activating the NF-κB pathway. Finally, the disturbed mTEC development and TRA expression influenced thymocyte development and tolerance induction, which finally led to spontaneous autoimmune phenotype in *Dhx9* cKO mice.
